# Supplementary material for: Shallow distance-dependent triplet energy migration mediated by endothermic charge-transfer
Source: Nat Commun. 2021 Mar 9;12:1532. doi: 10.1038/s41467-021-21561-1 (PMC7943758; doi:10.1038/s41467-021-21561-1)
Supplement: Supplementary file 1 — Supplementary Information [file 41467_2021_21561_MOESM1_ESM.pdf]

Supplementary Information for

Shallow distance-dependent triplet energy migration mediated by endothermic charge-transfer

Lai et al.

Supplementary Note 1. Electronic coupling matrix for direct triplet energy transfer

1.1 Dexter's formula

Following Dexter's formula,[1] in the weak coupling regime, we can use Fermi's golden rule to calculate the rate of triplet energy transfer from a photoexcited donor (D) to an acceptor (A):

$$k_{TET} = \frac{2\pi}{\hbar}\rho_{DA}|V|^2 = \frac{2\pi}{\hbar}\rho_{DA}\left|\left\langle \Psi_f \left| \frac{e^2}{\vec{r}_{12}} \right| \Psi_i \right\rangle\right|^2 \qquad (S1),$$

where $\hbar$ is the Planck constant, ρ_{DA} is the joint density of states, V is the matrix element of the perturbation between the initial state Ψ_i and the final state Ψ_f, and $\frac{e^2}{\vec{r}_{12}}$ is the two-electron operator. In the initial state, the donor (D) is in the spin-triplet excited state and the acceptor (A) is in its ground state; in the final state, the donor is in its ground state and the acceptor is in its triplet excited state.

Taking into consideration the indistinguishablity of the two electrons (1 and 2) involved in triplet energy transfer, the wavefunctions Ψ_i and Ψ_f can be expressed as:

$$\Psi_i = \frac{1}{\sqrt{2}}\left(\varphi_D^*(1)\varphi_A(2) - \varphi_D^*(2)\varphi_A(1)\right) \qquad (S2),$$

$$\Psi_f = \frac{1}{\sqrt{2}}\left(\varphi_D(1)\varphi_A^*(2) - \varphi_D(2)\varphi_A^*(1)\right) \qquad (S3),$$

where φ_D and φ_A are the wavefunctions of donor and acceptor, respectively, and the asterisks denote that D or A is in its excited state. Therefore, the electronic coupling matrix element is:

$$V = \left\langle \Psi_f \left| \frac{e^2}{\vec{r}_{12}} \right| \Psi_i \right\rangle$$

$$= \frac{1}{2}\left\langle \varphi_D(1)\varphi_A^*(2) + \varphi_D(2)\varphi_A^*(1) \middle| \frac{e^2}{\vec{r}_{12}} \middle| \varphi_D^*(1)\varphi_A(2) + \varphi_D^*(2)\varphi_A(1) \right\rangle$$

$$= \left\langle \varphi_D(1)\varphi_A^*(2) \middle| \frac{e^2}{\vec{r}_{12}} \middle| \varphi_D^*(1)\varphi_A(2) \right\rangle - \left\langle \varphi_D(1)\varphi_A^*(2) \middle| \frac{e^2}{\vec{r}_{12}} \middle| \varphi_D^*(2)\varphi_A(1) \right\rangle = J - K \quad (S4).$$

J and *K*, by definition, are the Coulomb and exchange integrals, respectively.

If we separate the wavefunctions into the electronic and spin parts and use the single-particle representation (HOMO and LUMO) for the electronic part of the wavefunctions, *J* and *K* can be further derived as:

$$J \equiv \left\langle \varphi_D(1)\varphi_A^*(2) \middle| \frac{e^2}{\vec{r}_{12}} \middle| \varphi_D^*(1)\varphi_A(2) \right\rangle$$

$$= \left\langle \Psi_{HO}^D(1)\Psi_{LU}^A(2) \middle| \frac{e^2}{\vec{r}_{12}} \middle| \Psi_{LU}^D(1)\Psi_{HO}^A(2) \right\rangle \langle \chi_D(1)|\chi_D^*(1)\rangle\langle \chi_A^*(2)|\chi_A(2)\rangle \qquad (S5),$$

$$K \equiv \left\langle \varphi_D(1)\varphi_A^*(2) \middle| \frac{e^2}{\vec{r}_{12}} \middle| \varphi_D^*(2)\varphi_A(1) \right\rangle$$

$$= \left\langle \Psi_{HO}^D(1)\Psi_{LU}^A(2) \middle| \frac{e^2}{\vec{r}_{12}} \middle| \Psi_{LU}^D(2)\Psi_{HO}^A(1) \right\rangle \langle \chi_D(1)|\chi_A(1)\rangle\langle \chi_A^*(2)|\chi_D^*(2)\rangle \qquad (S6).$$

For triplet energy transfer, *J* vanishes because of the different spin multiplicities of the excited and ground states of the donor (acceptor). Therefore, the electronic coupling matrix element is dominated by the *K* term and, by dropping off the operator, we can derive the following approximate expressions for V_{TET} and k_{TET}:

$$V_{TET} = K = \left\langle \Psi_{HO}^D\Psi_{LU}^A \middle| \frac{e^2}{\vec{r}_{12}} \middle| \Psi_{LU}^D\Psi_{HO}^A \right\rangle \propto \langle \Psi_{HO}^D|\Psi_{HO}^A\rangle\langle \Psi_{LU}^A|\Psi_{LU}^D\rangle \qquad (S7),$$

$$k_{TET} \propto |V_{TET}|^2 = K^2 \propto \langle \Psi_{HO}^D|\Psi_{HO}^A\rangle^2\langle \Psi_{LU}^A|\Psi_{LU}^D\rangle^2 \qquad (S8).$$

When the donor is a semiconductor QD, $\langle \Psi_{LU}^A|\Psi_{LU}^D\rangle^2$ and $\langle \Psi_{HO}^D|\Psi_{HO}^A\rangle^2$ are proportional to electron and hole probability densities on the QD surfaces ($|\Psi_e^S|^2$ and

$\left|\Psi_h^S\right|^2$), respectively. Therefore, the rate of direct TET should scale as:

$$k_{TET} \propto |V_{TET}|^2 \propto |\Psi_e^S|^2 \left|\Psi_h^S\right|^2 \qquad (S9).$$

1.2 Harcourt et al.'s formula

Harcourt et al. have reformulated the electronic coupling matrix element for excitation energy transfer by including through-configuration terms.[2] They find that the coupling matrix element, in the case of triplet energy transfer, can be approximated as:

$$V_{TET} \approx -K + \frac{T_{ET}T_{HT}}{A_{ET}} + \frac{T_{HT}T_{ET}}{A_{HT}} \qquad (S10),$$

where the two-electron exchange term K is the same as defined above. The second term is a through-configuration term resulting from a virtual electron transfer pathway, with T_{ET} and T_{HT} standing for the matrix elements of virtual electron transfer and the accompanying hole transfer, respectively, and A_{ET} is the energy difference between the virtual electron transfer state and the initial state. Similarly, the third term is the other through-configuration term resulting from a virtual hole transfer pathway. Using an ethene sandwich dimer as a model system, Scholes et al. have shown that the through-configuration terms can dominate over the two-electron exchange term K,[3] thus challenging conventional understanding for Dexter-type energy transfer processes.

For the QD-molecule donor-acceptor systems studied herein, because of the difficulty to compute the absolute values for the various terms defined in eq. S10, it remains unclear how the through-configuration terms compare with K. Nonetheless, because T_{ET} and T_{HT} should scale with Ψ_e^S and Ψ_h^S, respectively, the scaling relationship in eq. S9 should still hold.

Supplementary Note 2. Electronic coupling matrix for charge-transfer mediated triplet energy transfer

Charge-transfer (CT) mediated TET involves sequential transfer of an electron ad a hole from the donor to the acceptor. In this case, using the above procedures, it is easy to show that the rates of electron (k_{ET}) and hole transfer (k_{HT}) processes scale as :

$$k_{ET} \propto |V_{ET}|^2 \propto \langle \Psi_{LU}^{A} | \Psi_{LU}^{D} \rangle^2 \qquad (S11),$$

$$k_{HT} \propto |V_{HT}|^2 \propto \langle \Psi_{HO}^{D} | \Psi_{HO}^{A} \rangle^2 \qquad (S12).$$

When the donor is a QD, these scaling relationships can be further simplified:

$$k_{ET} \propto |V_{ET}|^2 \propto |\Psi_{e}^{S}|^2 \qquad (S13),$$

$$k_{HT} \propto |V_{HT}|^2 \propto \left|\Psi_{h}^{S}\right|^2 \qquad (S14).$$

In order to derive how the overall, apparent TET rate scales with QD wavefunctions, we consider the following coupled kinetic reaction:

$$QD^{*} - A \xleftrightarrow{k_1(k_{-1})} CT \overset{k_2}{\rightarrow} QD - A^{*} \qquad (S15),$$

where QD^{*}-A and QD-A^{*} are the initial and final states, respectively, CT is intermediate charge-transfer state, k_1 and k_2 are electron and hole transfer rates, and k_{-1} is the backward reaction rate of the first step. As we are dealing with the situation that CT is an endothermic state with higher energy than QD^{*}-A, the formation of CT via k_1 should be much slower compared to its decay via k_{-1} and k_2. Thus, the concentration of CT should always be low (undetectable on TA spectra in our experiment), allowing us to apply the steady-state approximation to solve the kinetic equations:

$$\frac{d[CT]}{dt} = k_1[QD^{*} - A] - k_{-1}[CT] - k_2[CT] = 0 \qquad (S16).$$

Therefore,

$$\frac{d[QD-A^*]}{dt} = k_2[CT] = \frac{k_1 k_2 [QD^* - A]}{k_{-1} + k_2} \qquad (S17),$$

and the apparent rate for the formation of acceptor triplets is:

$$k_{TET} \approx \frac{k_1 k_2}{k_{-1} + k_2} \qquad (S18).$$

Under the situation that k_2 are much faster than k_1 and k_{-1} (as is in our case), the overall TET rate is reduced to k_1 and it should simply scale as $|\Psi_e^S|^2$ or $|\Psi_h^S|^2$ depending on whether the first step is an electron or hole transfer process.

Supplementary Note 3. Calculations of carrier wavefunctions in QDs

The probability densities (squared wavefunctions) of the band edge electron and hole of CdSe/ZnS QDs were simulated using a single-band effective mass approximation model (EMA). The Coulomb interaction between the electron and the hole was also ignored in our simulation but can be readily added using first-order perturbation when calculating optical energy gaps.

A model of a particle in 3-region spherical potentials is employed. The three regions from the center to the outside of the sphere correspond to the CdSe core, ZnS shell and oleate ligand layer, and their radius or thickness are defined as the radius of CdSe core size, ZnS shell thickness (0.27 nm per monolayer multiplied by the layer numbers) and 1.5 nm, respectively. Thus, the potentials were set as:

$$\mathrm{V}(r) = \begin{cases} 0, & 0 \le r \le R_{core} \\ E_{CB,ZnS}\ (or\ E_{VB,ZnS}), & \mathrm{R}_{core} \le r \le \mathrm{R}_{core/shell} \\ E_{LUMO,OA}\ (or\ E_{HOMO,OA}), & \mathrm{R}_{core/shell} \le r \le \mathrm{R}_{core/shell} + 1.5 \end{cases} \qquad (S19),$$

in which R_{core} stands for the radius CdSe core QDs in the unit of nm (which is 1.2

nm in our case), $R_{core/shell}$ is the radius of CdSe/ZnS QDs, $E_{CB,ZnS}$ ($E_{VB,ZnS}$) is the energy offset between the CBMs (VBMs) of bulk ZnS and CdSe, and $E_{LUMO,OA}$ ($E_{HOMO,OA}$) is that between the LUMO (HOMO) of oleic acid and the CBM (VBM) of bulk CdSe. The CBM and VBM of bulk CdSe are both set to 0 in the simulation. The effective mass of the electron (hole) in the above-mentioned three regions was defined as:

$$m^*_{e(h)}(r) = \begin{cases} m^*_{e(h),CdSe}, 0 \le r \le R_{core} \\ m^*_{e(h),ZnS}, \mathrm{R}_{core} \le r \le \mathrm{R}_{core/shell} \\ m_0, \mathrm{R}_{core/shell} \le r \le \mathrm{R}_{core/shell} + 1.5 \end{cases} \qquad (S20),$$

where m_0 is the free electron mass and $m^*_{e(h),CdSe}$ and $m^*_{e(h),ZnS}$ are the electron (hole) effective mass in bulk CdSe and ZnS, respectively.

The parameters used in the calculations are summarized in Supplementary Table 1. The simulation was performed in COMSOL Multiphysics® Modeling Software using the semiconductor module. The carrier probability densities on QD surfaces were the average values over the surface areas of the QDs.

Supplementary Table 1. Parameters for EMA wavefunction calculations.

	m_e* (m_0)	m_h* (m_0)	CBM/LUMO (eV vs. vac.)	VBM/HOMO (eV vs. vac.)
CdSe[4,5]	0.12	0.40	-4	-5.75
ZnS[4]	0.28	0.50	-3	-6.6
OA[6]	1	1	-0.7	-6.8

Supplementary Note 4. Estimation of TET and CT driving forces

4.1. Redox potential energies of ACA

The oxidation potential energy of ground state ACA ($E_{ACA^+/ACA}$) can be determined from the CV measurement using the ferrocene/ferrocenium (Fc/Fc^+) pair as a reference (**Supplementary Figure**). The absolute energy of Fc/Fc^+ is -4.84 eV vs vacuum in the solvent used here (dichloromethane/acetonitrile = 4:1).[7] $E_{ACA^+/ACA}$ can be calculated as:

$$E_{ACA^+/ACA} = -\left(\frac{E_{ap,ACA}+E_{cp,ACA}}{2} - \frac{E_{ap,Fc}+E_{cp,Fc}}{2} + 4.84\right)$$
$$= -\left(\frac{1.66 + 1.385}{2} - \frac{0.747 + 0.341}{2} + 4.84\right) = -5.82\ eV \qquad (S21).$$

The oxidation potential energy of ACA triplet excited state ($E_{ACA^+/\,^3ACA^*}$) is:

$$E_{ACA^+/\,^3ACA^*} = E_{ACA^+/ACA} + E_T = -5.82 + 1.83 = -3.99\ eV \qquad (S22),$$

where E_T is the triplet energy of ACA which is 1.83 eV.

The oxidation potential energy of ACA singlet excited state ($E_{ACA^+/\,^1ACA^*}$) is:

$$E_{ACA^+/\,^1ACA^*} = E_{ACA^+/ACA} + E_S = -5.82 + 3.10 = -2.72\ eV \qquad (S23),$$

where E_S is the singlet energy of ACA which is determined as 3.1 eV from the cross point of normalized absorption and emission spectra.

The reduction potential energy of ground state ACA ($E_{ACA^-/ACA}$) is:

$$E_{ACA^-/ACA} = E_{ACA^+/\,^1ACA^*} + E_{b,ACA} = -2.72 + 0.8 = -1.92\ eV \qquad (S24),$$

where $E_{b,ACA}$ is exciton binding energy in ACA which is reported to be ~0.8 eV.[8] Note that all the Coulombic energy terms are defined as positive values here.

4.2. Redox potential energies of QDs

The oxidation potential energy of ground state CdSe/1.2ZnS QDs ($E_{QD^+/QD}$) is revealed as an anodic peak in **Supplementary Figure** . Unlike molecules, this peak is irreversible for QDs.[9] Therefore the anodic peak of ACA^+/ACA pair is used as a new reference:

$$E_{QD^+/QD} = -\left(\left(E_{ap,QD} - \frac{E_{ap,Fc} + E_{cp,Fc}}{2} + 4.86\right) - \left(E_{ap,ACA} - \frac{E_{ap,Fc} + E_{cp,Fc}}{2} + 4.84\right) + 5.82\right)$$
$$= -\left(\left(1.67 - \frac{0.685 + 0.211}{2} + 4.86\right) - \left(1.66 - \frac{0.747 + 0.341}{2} + 4.84\right) + 5.82\right) = -5.94\ eV\ (S25).$$

The oxidation potential energy of QD excited state ($E_{QD^+/QD*}$) is:

$$E_{QD^+/QD^*} = E_{QD^+/QD} + E_{g,QD} = -5.94 + 2.41 = -3.53\ eV \qquad (S26),$$

where $E_{g,QD}$ is the optical gap of QDs which is ~2.41 eV (515 nm). Note that, because of a weak exchange splitting in QDs (<20 meV),[10] the singlet and triplet excited state energies of QDs are assumed to be the same.

The reduction potential energy of ground state QDs ($E_{QD^-/QD}$) is:

$$E_{QD^-/QD} = E_{QD^+/QD^*} + E_b = -3.53 + 0.2 = -3.33\ eV \qquad (S27),$$

where $E_{b,QD}$ is exciton binding energy in QD which is estimated to be ~0.2 eV for CdSe QDs with the lowest absorption peak at ~515 nm using the model by Brus[11].

The reduction potential energy of QD excited state ($E_{QD^-/QD*}$) is:

$$E_{QD^-/QD^*} = E_{QD^-/QD} - E_{g,QD} = -3.33 - 2.41 = -5.74\ eV \qquad (S28).$$

4.3. TET and CT driving forces

Using the state potential energies determined above, rather than commonly-adopted single-particle potential energies (such as HOMO and LUMO for molecules and electron and hole levels for QDs), allows us to facilely calculate the free energy changes (i.e., driving forces) from reactant states to product states, because the electron correlation and exchange energy terms are already included in the state potential energies. An additional term that needs to be added, when applicable, is the Coulomb binding energy of the charge separated states.

For direct TET from photoexcited QDs to ACA, the free energy change can be written as:

$$\Delta G_{TET} = E_T - E_{g,QD} = 1.83 - 2.41 = -0.58\, eV \qquad (S29),$$

where E_T and $E_{g,QD}$ are the triplet energy of ACA and optical gap of QDs, respectively, as defined above.

For hole transfer from photoexcited QDs to ACA, the free energy change can be written as:

$$\Delta G_{HT} = E_{QD^-/QD^*} - E_{ACA^+/ACA} - E_{cs} = -5.74 + 5.82 - 0.05 = 0.03 eV \qquad (S30).$$

Here E_{cs} is the stabilization energy resulting from the above-mentioned charge separated state (QD^--ACA^+) binding energy, which can be estimated as ~50 meV for CdSe/1.2ZnS QDs using the Gauss's theorem. Therefore, this hole transfer process is energetically uphill by ~0.03 eV.

For electron transfer from photoexcited QDs to ACA, the free energy change can be written as:

$$\Delta G_{ET} = E_{ACA^-/ACA} - E_{QD^+/QD^*} - E_{cs} = -1.92 + 3.53 - 0.05 = 1.56\, eV \qquad (S31).$$

This electron transfer process is strongly disallowed.

There is another electron transfer process that is involved in endothermic hole-transfer-mediated triplet migration, electron transfer from QD^- to ACA^+ to form $^3ACA^*$. The free energy change of this process can be written as:

$$\Delta G_{ET2} = E_{ACA^+/\ ^3ACA^*} - E_{QD^-/QD} + E_{cs} = -3.99 + 3.34 + 0.05 = -0.6\ eV \quad (S32).$$

We note that all the calculations above are made for CdSe/1.2ZnS QDs for which the CV measurement was performed. We assume that QDs of other shell thicknesses have very similar TET or CT driving forces for the following reasons. First, the changes in exciton binding energy and QD charging energy with shell thickness almost mutually cancels out, as evidenced by the negligible exciton absorption peak shift from the thinnest shell CdSe/0.5ZnS QDs to the thickest shell CdSe/3.9ZnS QDs. Second, according to our calculation, the E_{cs} term changes by less than 30 meV from the thinnest to thickest shell QDs. Thus, TET and CT driving forces can be treated as shell-thickness-independent in our experiments.

Supplementary Note 5. PL kinetics analysis

PL kinetics were monitored at peak wavelengths of PL peaks. The overall PL kinetics were obtained by combining femtosecond fluorescence upconversion and TCSPC decay curves. The kinetic curves of free QDs and QD-ACA complexes could be well fitted by multiple-exponential functions with 2 or 3 components:

$$I_{QD}(t) = \sum_{i=1}^{n} A_{QD,i} \exp\left(-\frac{t}{\tau_{QD,i}}\right) \quad (S33),$$

$$I_{QD-ACA}(t) = \sum_{i=1}^{n} A_{QD-\mathrm{ACA},i} \exp\left(-\frac{t}{\tau_{QD-ACA,i}}\right) \quad (S34).$$

The average lifetime was calculated as:

$$\langle \tau_{QD} \rangle = \frac{\sum_{i=1}^{n} A_{QD,i} \tau_{QD,i}}{\sum_{i=1}^{n} A_{QD,i}} \qquad (S35),$$

$$\langle \tau_{QD-ACA} \rangle = \frac{\sum_{i=1}^{n} A_{QD-ACA,i} \tau_{QD-ACA,i}}{\sum_{i=1}^{n} A_{QD-ACA,i}} \qquad (S36).$$

Triplet energy transfer quantum yield ϕ_{TET} is defined as:

$$\phi_{TET} = \frac{k_{QD-ACA} - k_{QD}}{k_{QD-ACA}} = 1 - \frac{\langle \tau_{QD-ACA} \rangle}{\langle \tau_{QD} \rangle} \qquad (S37).$$

The average transfer energy transfer rate from QD to each anchored ACA molecule is calculated as:

$$\langle k_{TET} \rangle = \frac{\frac{1}{\langle \tau_{QD-ACA} \rangle} - \frac{1}{\langle \tau_{QD} \rangle}}{n_{ACA}} \qquad (S38).$$

in which n_{ACA} stands for the average number of anchored ACA molecules per QD. The kinetics fitting parameters and calculated energy transfer yields and rates are listed in Supplementary Table 2.

Supplementary Table 2. Multi-exponential fitting of TR-PL kinetics of free QDs and QD-ACA complexes.

	A_1	$\tau_1\ (ps)$	A_2	$\tau_2\ (ps)$	A_3	$\tau_3\ (ps)$	n_{ACA}	$\langle\tau\rangle\ (ps)$	ϕ_{TET}	$k_{TET}\ (ns^{-1})$
CdSe/0.5ZnS	85	1135	323.1	21965	/	/	/	17626.48	/	/
CdSe/0.5ZnS-ACA	148	155	49	2511.9	33	15460	20.8	2853.06	0.838	0.014123
CdSe/1.2ZnS	205	746.3	565	22255	/	/	/	16528.66	/	/

CdSe/1.2ZnS-ACA	230	163	156	2553.8	198	17075	20.9	6535.50	0.605	0.004426
CdSe/1.7ZnS	134	1085	713	20912	/	/	/	17775.26	/	/
CdSe/1.7ZnS-ACA	266	292	156	3369	344	17805	25.2	8783.49	0.506	0.002285
CdSe/2.2ZnS	137	150	101	3473	532	19898	/	14229.95	/	/
CdSe/2.2ZnS-ACA	121	150	148	3188	325	17336	32.6	10310.06	0.275	0.00082
CdSe/2.4ZnS	57	1682	1517	22113	/	/	/	21373.12	/	/
CdSe/2.4ZnS-ACA	382.2	3049	1104	19291	/	/	34.1	15114.11	0.293	0.000568
CdSe/2.8ZnS	255.7	408.6	919.5	22105	/	/	/	17384.30	/	/
CdSe/2.8ZnS-ACA	143	217	130	3081	668	19681	33.3	14429.83	0.170	0.000354
CdSe/3.9ZnS	73	1280	778	21291	/	/	/	19574.43	/	/
CdSe/3.9ZnS-ACA	111	2531	691	19855	/	/	52.2	17457.29	0.108	0.000119

Supplementary Note 6. Kinetic simulation

We simulated the temporal evolution of QD^*-ACA, QD^--ACA^+ (i.e. CT state) and QD-$^3ACA^*$ species using the coupled rate equations of eqs. S39-41, where k_1 and k_2 are hole and electron transfer rates, respectively, and k_{-1} is the backward hole transfer rate. The value of k_1 was from the experimental data, whereas k_2 and k_{-1} were estimated according the Marcus equation by assuming that the reorganization energies were the same for these CT processes.

$$\frac{d[QD^* - A]}{dt} = -k_1[QD^* - A] + k_{-1}[CT] - k_r[QD^* - A] \quad (S39)$$

$$\frac{d[CT]}{dt} = k_1[QD^* - A] - (k_{-1} + k_2)[CT] \quad (S40)$$

$$\frac{d[QD - {}^3A^*]}{dt} = k_2[CT] \quad (S41)$$

We chose CdSe/0.5ZnS QD-ACA complexes for the simulation, because the ratio between k_1 and k_2 is largest for this sample, i.e. the most possible one for us to detect the ACA cations. The temporal evolution of the transient species is presented in Supplementary Fig. 10. From the simulation, the QD^--ACA^+ CT species contributes at most 1% of the total species population at time delay of 0.1-0.2 ns. Further considering the orders-of -magnitude difference in the extinction coefficients of QDs and ACA cations, it is technically impossible to detect the ACA cations on TA spectra.

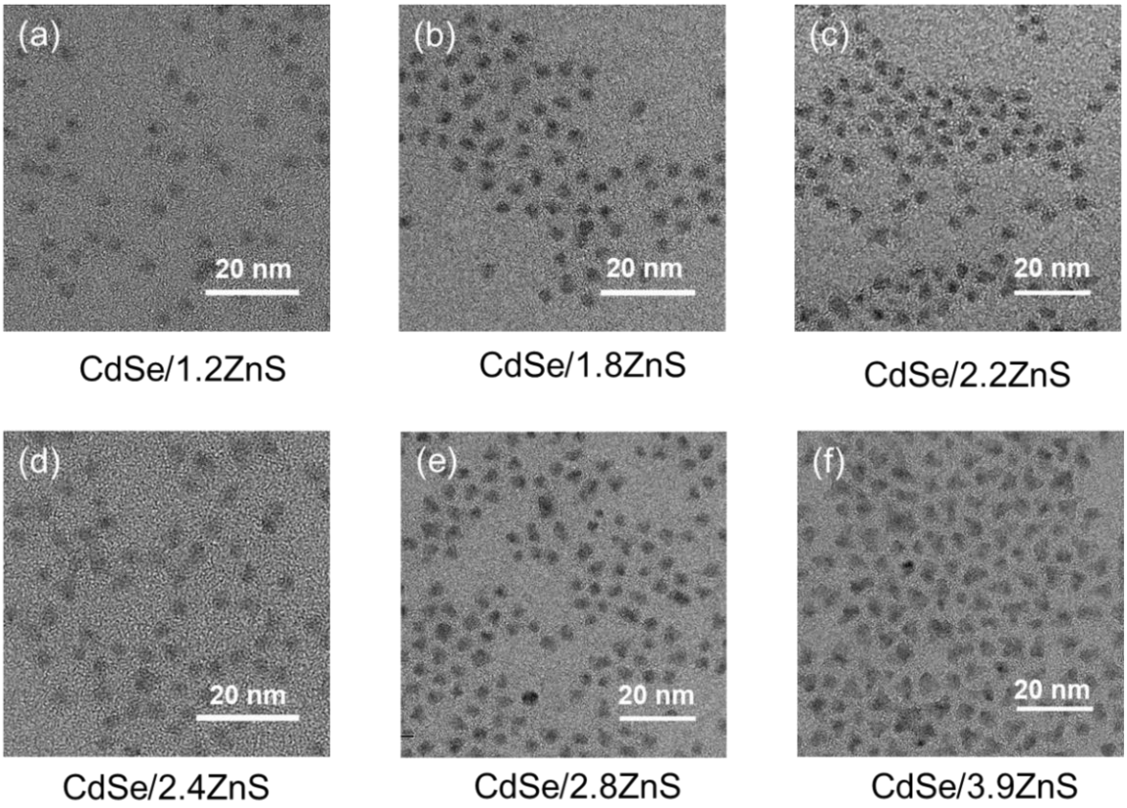

Supplementary Figure 1. **TEM images.** Representative TEM images of CdSe/ZnS QDs with different ZnS shell thicknesses. (a) CdSe/1.2ZnS. (b) CdSe/1.8ZnS. (c) CdSe/2.2ZnS. (d) CdSe/2.4ZnS. (e) CdSe/2.8ZnS. (f) CdSe/3.9ZnS.

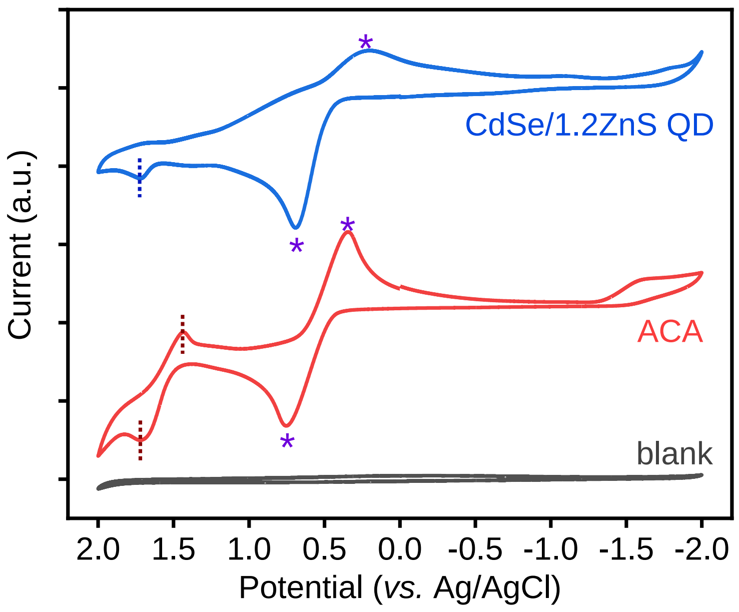

Supplementary Figure 2. CV measurements. Cyclic voltammetry curves of CdSe/1.2ZnS QDs and ACA molecules. The anodic peaks for the oxidation of ACA and QDs are at very similar potentials.

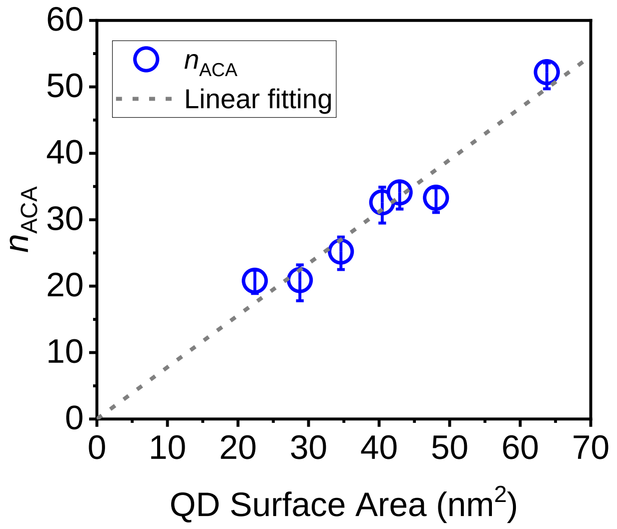

Supplementary Figure 3. **Number of ACA molecules.** Average ACA number per QD (n_{ACA}) versus the surface area of QD in QD-ACA complexes with different ZnS shell thicknesses. Grey dash line is a linear fit. Error bars are the standard deviations for calculations using the absorption intensities and extinction coefficients of ACA at its three vibronic peaks.

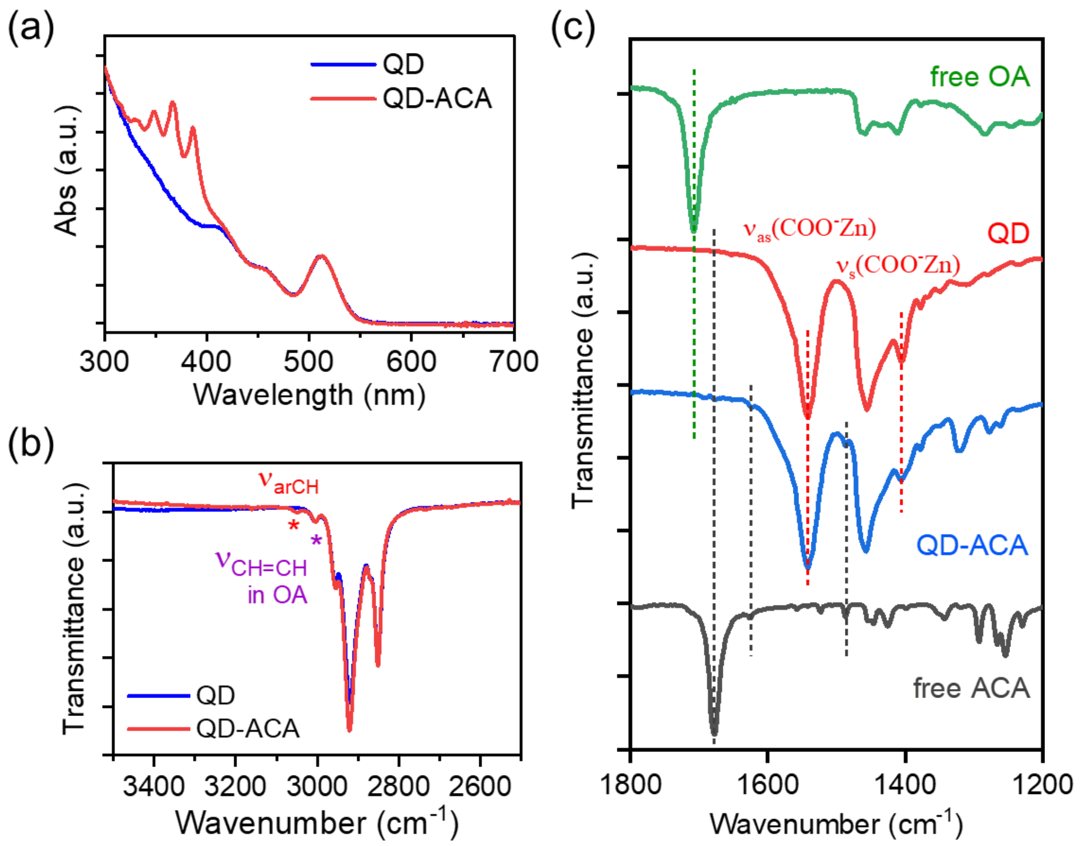

Supplementary Figure 4. QD surface analysis. (a) Absorption spectra of CdSe/1.2ZnS QDs and their -ACA complexes used in Fourier transform infrared spectroscopy (FTIR) and gas chromatography–mass spectrometry (GC-MS) measurements. (b) FTIR spectra of QD and QD-ACA complexes in region of 2500-3500 cm^{-1}, showing additional stretching vibration signatures of aromatic C-H bonds in ACA molecules. (c) Comparison of FTIR spectra of free oleic acid, free ACA, QD and QD-ACA complexes in an enlarged view of 1200-1800 cm^{-1}. Dash lines indicate characteristic peaks of carboxylic groups and zinc carboxylate. No free oleic acid or ACA features can be observed for the QD-ACA sample, indicating that

ACA binds to unoccupied sites on QD surfaces (i.e., not by replacing native oleic acid ligands). To verify this, we used GC-MS to quantify that there were ~23 ACA per QD in the QD-ACA sample, which is consistent with the number estimated from the absorption spectra. Also, there were 47-75 oleic acid ligands per QD in both free QD and QD-ACA samples. This ligand density corresponds to a ligand-to-ligand distance of 0.6-0.8 nm, which should allow for ACA insertion between the ligands.

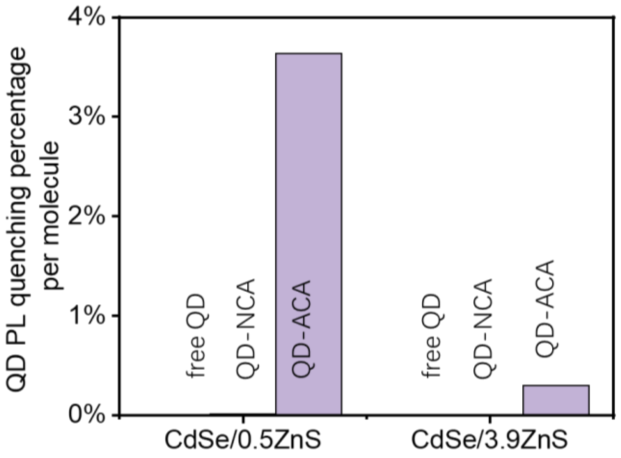

Supplementary Figure 5. **PL quenching induced by ligands.** PL quenching of CdSe/ZnS QDs with anchored ACA or NCA molecules. The excitation wavelengths are both set at 460 nm to avoid direct excitation of molecules. The PL quenching capability is normalized to each anchored molecule. CdSe/0.5ZnS and CdSe/3.9ZnS QDs are presented.

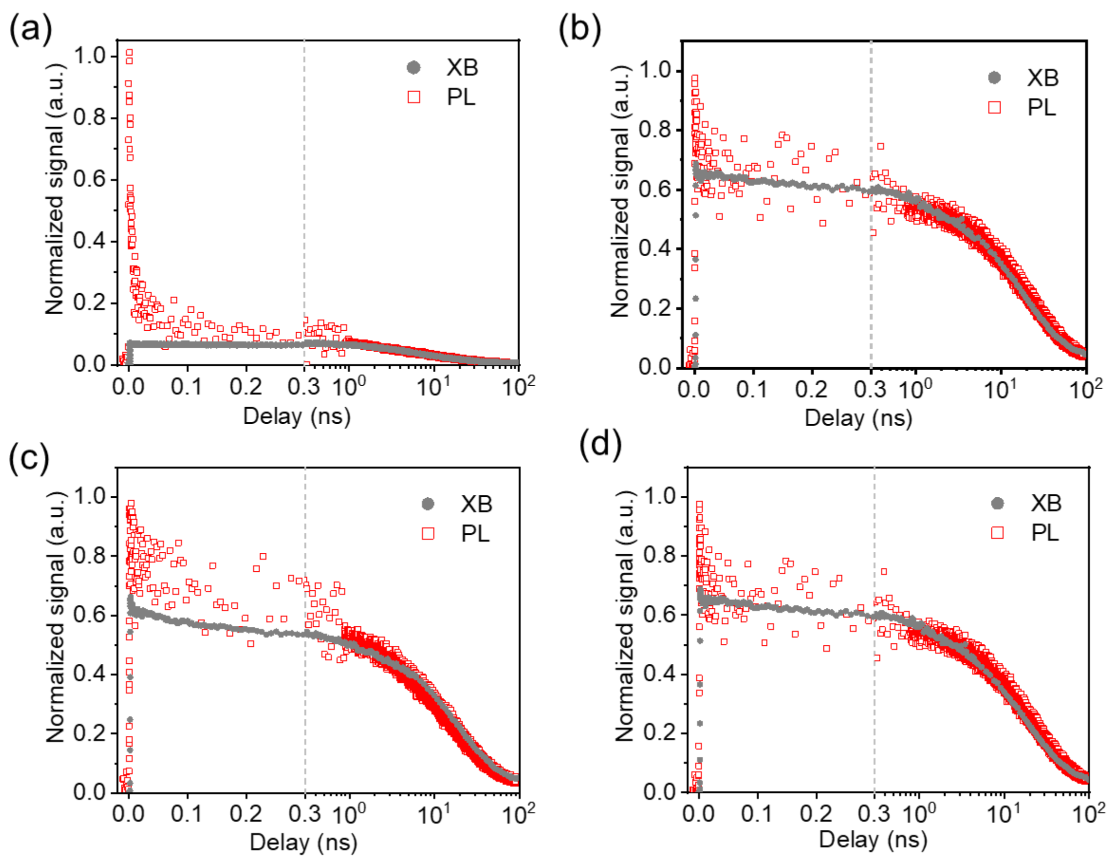

Supplementary Figure 6. **Hole trapping in QDs.** Comparison of the TA exciton bleach (XB) kinetics and time-resolved PL kinetics of (a) CdSe core, (b) CdSe/0.5ZnS, (c) CdSe/2.2ZnS, and (d) CdSe/2.8 ZnS free QDs. The strong difference between XB and PL kinetics in core-only QDs is caused by hole trapping on the ps time scale that can be effectively alleviated in core/shell QDs.

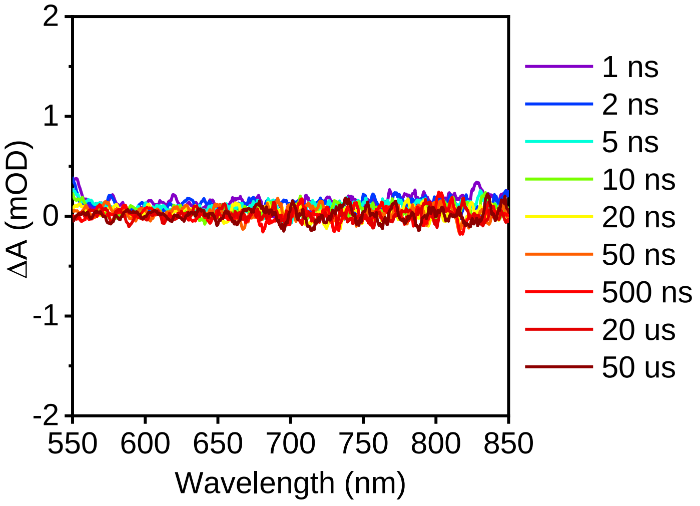

Supplementary Figure 7. **The absence of ACA anions or cations.** TA spectra of CdSe/1.2ZnS QD-ACA complexes in the spectral region of 550-850 nm showing no signature of ACA anion or cation radicals.

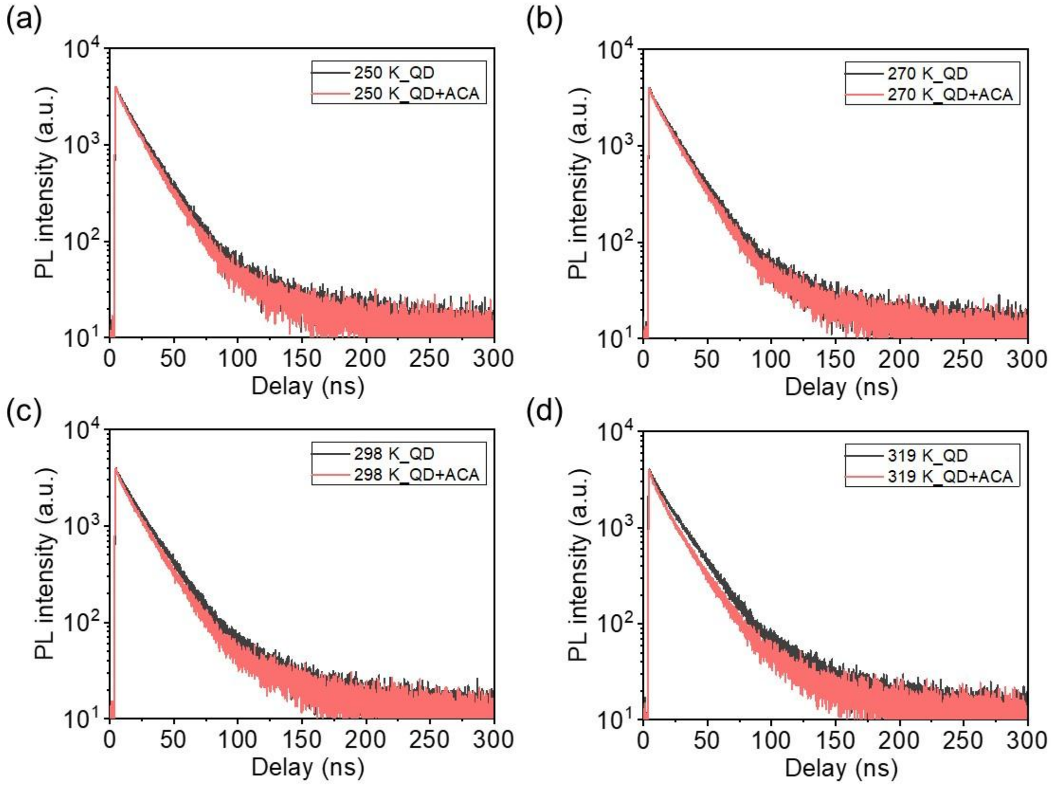

Supplementary Figure 8. **Temperature dependent TR-PL kinetics.** Representative temperature dependent TR-PL traces at (a) 250 K, (b) 270 K, (c) 298 K and (d) 319 K. PL decays of free CdSe/2.2ZnS QDs (black lines) and their -ACA complexes (red lines) in hexane.

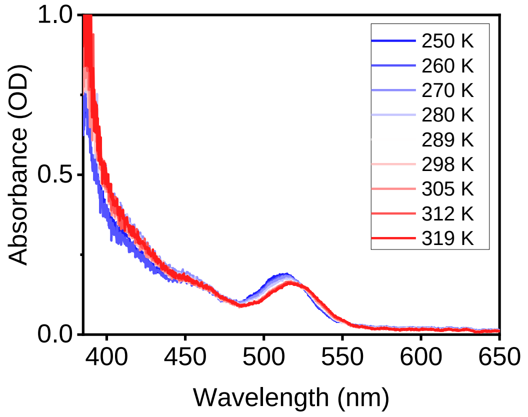

Supplementary Figure 9. **Temperature dependent absorption spectra.** Temperature dependent absorption spectra of CdSe/2.2ZnS QDs showing blue-shift of peaks upon decreasing temperature.

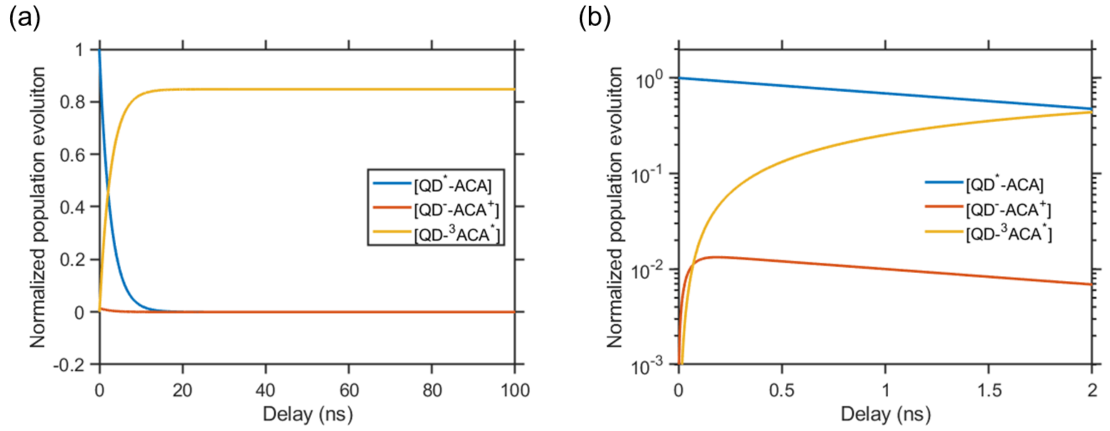

Supplementary Figure 10. **Kinetics simulation.** Simulated temporal evolution of relative populations of QD*-ACA, QD^{-}-ACA^{+} and QD-$^{3}ACA^{*}$ in CdSe/0.5ZnS-ACA complexes in the first (a) 100 ns and (b) 2 ns.

Supplementary References

1 Dexter, D. L. A Theory of Sensitized Luminescence in Solids. *J. Chem. Phys.* **21**, 836-850 (1953).

2 Harcourt, R. D., Scholes, G. D. & Ghiggino, K. P. Rate expressions for excitation transfer. II. Electronic considerations of direct and through–configuration exciton resonance interactions. *J. Chem. Phys.* **101**, 10521-10525 (1994).

3 Scholes, G. D., Harcourt, R. D. & Ghiggino, K. P. Rate expressions for excitation transfer. III. An ab initio study of electronic factors in excitation transfer and exciton resonance interactions. *J. Chem. Phys.* **102**, 9574-9581 (1995).

4 Liu, E. *The Physics of Semiconductors*. 7th edn, (Publishing House of Electronics Industry, 2008).

5 Zhu, H. M., Yang, Y., Hyeon-Deuk, K., Califano, M., Song, N. H., Wang, Y. W., Zhang, W. Q., Prezhdo, O. V. & Lian, T. Q. Auger-Assisted Electron Transfer from Photoexcited Semiconductor Quantum Dots. *Nano Lett.* **14**, 1263-1269 (2014).

6 Valencia, D., García-Cruz, I., Uc, V. H., Ramírez-Verduzco, L. F., Amezcua-Allieri, M. A. & Aburto, J. Unravelling the chemical reactions of fatty acids and triacylglycerides under hydrodeoxygenation conditions based on a comprehensive thermodynamic analysis. *Biomass and Bioenergy* **112**, 37-44 (2018).

7 Connelly, N. G. & Geiger, W. E. Chemical Redox Agents for Organometallic

Chemistry. *Chem. Rev.* **96**, 877-910 (1996).

8 Hummer, K., Puschnig, P. & Ambrosch-Draxl, C. Lowest Optical Excitations in Molecular Crystals: Bound Excitons versus Free Electron-Hole Pairs in Anthracene. *Phys. Rev. Lett.* **92**, 147402 (2004).

9 Haram, S. K., Quinn, B. M. & Bard, A. J. Electrochemistry of CdS Nanoparticles: A Correlation between Optical and Electrochemical Band Gaps. *J. Am. Chem. Soc.* **123**, 8860-8861 (2001).

10 Efros, A. L. & Rosen, M. The Electronic Structure of Semiconductor Nanocrystals. *Annu. Rev. Mater. Sci.* **30**, 475-521 (2000).

11 Brus, L. E. Electron--electron and electron-hole interactions in small semiconductor crystallites: The size dependence of the lowest excited electronic state. *J. Chem. Phys.* **80**, 4403-4409 (1984).

12 Rurack, K. & Spieles, M. Fluorescence Quantum Yields of a Series of Red and Near-Infrared Dyes Emitting at 600-1000 nm. *Anal Chem* **83**, 1232-1242 (2011).
